# Study of Hydraulic Properties of Uncoated Paper: Image Analysis and Pore-Scale Modeling

**DOI:** 10.1007/s11242-017-0909-x

**Published:** 2017-08-03

**Authors:** H. Aslannejad, S. M. Hassanizadeh

**Affiliations:** 1 Department of Earth Sciences, Utrecht University, Utrecht, The Netherlands; 2 Environmental Hydrogeology Group, Universiteit Utrecht, Princetonplein 9, 3584 Utrecht, The Netherlands

**Keywords:** Uncoated paper, 3D pore network, Image analysis, Pore-scale modeling, Water flow in fibrous porous media

## Abstract

In this study, uncoated paper was characterized. Three-dimensional structure of the layer was reconstructed using imaging results of micro-CT scanning with a relatively high resolution $(0.9~\upmu \hbox {m})$. Image analysis provided the pore space of the layer, which was used to determine its porosity and pore size distribution. Representative elementary volume (REV) size was determined by calculating values of porosity and permeability values for varying domain sizes. We found that those values remained unchanged for domain sizes of $400\times 400\times 150\,\upmu \hbox {m}^{3}$ and larger; this was chosen as the REV size. The determined REV size was verified by determining capillary pressure–saturation  imbibition curves for various domain sizes. We studied the directional dependence of  curves by simulating water penetration into the layer from various directions. We did not find any significant difference between  curves in different directions. We studied the effect of compression of paper on  curves. We found that up to 30% compression of the paper layer had very small effect on the  curve. Relative permeability as a function of saturation was also calculated. Water penetration into paper was visualized using confocal laser scanning microscopy. Dynamic visualization of water flow in the paper showed that water moves along the fibers first and then fills the pores between them.

## Introduction

Paper sheets and paper-based products like tissues and packing materials are commonly used in everyday life. Special fluid flow properties are required in such materials to achieve desired results; for instance, in the case of printing, penetration of ink into the paper affects the print quality significantly. Uncontrolled ink movement on/in paper layer potentially will cause poor print qualities like color bleeding and prints contamination on the other side of paper. So, in order to achieve the desired print quality, by controlling ink spreading and penetration, we need to understand ink flow inside the porous structure of paper. A valuable tool in this regard is the macroscale simulation of ink penetration into paper. For macroscale model of ink movement in paper, values of hydraulic properties, such as porosity, permeability, capillary pressure–saturation curves, and relative permeability curves, are needed. In the absence of appropriate experimental methods, we obtain values of hydraulic properties by means of image analysis and a pore-scale model.

Normal plain paper is a fibrous layer consisting of cellulose fibers, which form a special type of pore network (different from pore network of a granular porous medium). For a granular porous medium, there is a clear distinction between pore body and pore throat. A fibrous layer, however, has a complex structure with large and irregular shape pores with fiber’s surface as the solid part of domain. In order to model fluid flow inside such a material, we need to characterize the three-dimensional structure of the layer in detail. This is usually done by means of imaging techniques.

Various imaging techniques have been used for papers like: X-ray tomography, focused ion beam secondary electron microscopy, and confocal microscopy. Aronsson et al. (2001) acquired two-dimensional SEM images of cardboard paper. They reconstructed 3D structure of paper using a micro-tomography destructive method to slice layers of paper and acquired stacks of 2D images. Samuelsen et al. (2001) obtained 3D images of paper using nondestructive synchrotron X-ray micro-tomography. They used the images for the study of mechanical properties of paper. Rolland du Roscoat et al. (2008) also used synchrotron X-ray micro-tomography to study two different types of paper, Eucalyptus pulp handsheet and Blotting paper. They carried out pore-scale calculations and determined values of thermal conductivity and permeability. They also analyzed synchrotron results in order to determine the variation of porosity over the paper thickness, and checked anisotropy and heterogeneity of the microstructure. Holmstad (2005) did a comparison study on low- and high-resolution X-ray tomography results of paper samples for obtaining structural information. They concluded that high-resolution images are required for research into paper structure.

Axelsson (2009) demonstrated that digital image analysis of X-ray images is a fast, automatic, and reproducible method for characterizing microstructure of porous materials. In 2010, Axelsson and Svensson (2010) proposed a new computational method to extract pore network of duplex paper structure from scanned images. They introduced a pore-based skeleton representation of the material which is useful for permeability and tortuosity calculations.

Pore size distribution and porosity determination can be done either experimentally (mercury porosimetry) or by image analysis methods. In case of determination by image analysis, pore space of the domain should be reconstructed. Delerue et al. (1999) introduced a new skeletization method to obtain the pore space of a porous material from 3D images. Huang et al. (2002) developed a method for using X-ray micro-tomography to determine structural parameters like porosity, pore size distribution, and specific surface area of fibers. They concluded that their results compared favorably with mercury intrusion porosimetry results.

Performing direct numerical simulation on a big domain size usually is prohibitive because of computational cost. So, effective properties of a porous material should be determined for a minimum domain size which is Representative Elementary Volume (REV). The REV size can be determined by analyzing domain size influence on values of physical and hydraulic parameters (Ramaswamy et al. 2001). Kanit et al. (2003) proposed a REV determination method which is applicable to real three-dimensional images of heterogeneous materials obtained by imaging techniques. Ramaswamy et al. (2001) determined REV size for a glass bead packing by analyzing the change in porosity and specific surface area values with increasing domain size.

Several studies have been done to determine permeability values for paper samples. Lindsay (1988) conducted an experimental study of measuring paper permeability. They found that the anisotropy of fibrous layer has influence on permeability values and the ratio of lateral to transverse permeability is less than 2. Zhu et al. (1995) developed a mechanistic model to describe fluid permeation through fiber beds. Their method was based on the micro-mechanical theories of compressibility of fiber assemblies. First, they determined the porosity profile along thickness. Then, they related permeability to the porosity with help of the Davies–Ingmanson equation.

Das and Ramarao (2002) determined permeability and compressibility relation for filter cakes formed during cake filtration. They showed that permeability for pulp mats is related to the specific surface area and volume of fibers. Wei and Ramarao (1996) introduced a novel drainage test to determine specific surface area and volume of fibers which are required for permeability calculation.

Ingmanson et al. (1959) modified Kozeny–Carman equation for calculation of permeability value for paper containing fillers and pulp fibers in the following form:

1 $$\begin{aligned}&\displaystyle K=\frac{1}{kS_{w,f}^2 }\frac{\left( {1-v_{f,f} C} \right) ^{3}}{C^{2}} \end{aligned}$$

2 $$\begin{aligned}&\displaystyle C=\frac{m_f +m_p }{v_f +v_p +v_v } \end{aligned}$$

3 $$\begin{aligned}&\displaystyle S_{w,f} =S_{0,f} .v_{f,f} \end{aligned}$$

where *k* is an empirical constant, $$S_{0,f} $$ is specific surface area of fibers $$(\hbox {m}^{-1})$$, $$v_{f,f} $$ is specific volume of fibers $$(\hbox {m}^{3}\, \hbox {kg}^{-1})$$, $$m_f\,\mathrm{and}\,m_p$$ are masses of fibers and fillers, respectively, $$v_f ,v_p ,v_v $$ are volumes of fibers, fillers and void space $$(\hbox {m}^{3})$$, respectively.

In order to understand fluid flow inside a fibrous layer, one needs to know more about the structure of the material and its composition. Zhao et al. (2007) did a comprehensive study of cellulose fiber structure using SEM, XRD, NMR and acid hydrolysis. Based on their study, each cellulose fiber is composed of micro-fibril bundles (diameters of 20–30 nm). Hirn and Schennach (2015) found out that fibers in a paper bond to each other by six different mechanisms: interdiffusion, mechanical interlocking, capillary forces, Coulomb forces, hydrogen bonding, and van der Waals forces. They concluded that, in contrast to general belief that favors hydrogen bonding, van der Waals bonds play the most important role.

**Table 1:** Physical properties of paper samples$^\mathrm{a}$

Property	Thickness $(\upmu \hbox {m})$	Grammage (gsm)$^\mathrm{b}$	Smoothness$^\mathrm{c}$ ($\upmu \hbox {m}$)	Ash content (%)	Surface PH	Relative humidity$^\mathrm{d}$ (%)
Value	105	$90\pm 2$	$4.50\pm 0.5$	$12.0\pm 1$	$7.3\pm 0.5$	$40\pm 4$

$^\mathrm{a}$ Data were obtained from www.zieglerpapier.com

$^\mathrm{b}$ Gram per square meter

$^\mathrm{c}$ Measured by Parker Print-surf method which measures the roughness of paper and paperboard under conditions intended to simulate the press nip pressures and backing substrates found in printing processes

$^\mathrm{d}$ Product condition before experiment

In this paper, we report on a comprehensive study of pore space and hydraulic properties of an uncoated paper. Through analysis of high-resolution micro-computational tomography $(\upmu \hbox {CT})$ images, we obtained the pore structure of the fibrous layer. Then, pore space properties including porosity and pore size distribution were obtained. In addition, permeability, capillary pressure– saturation curves, and relative permeability curves were determined using pore morphology and solving Stokes and Darcy equations. The REV size for determining average properties of the layer was determined through investigation of porosity variation, anisotropic permeability, and capillary pressure curves for various domain sizes. Finally, in order to find out more about fluid flow in such a layer, an experimental visualization of ink movement in fibrous layer was performed and analyzed.

## Material and Methods

### Paper Sample

**Fig. 1:**
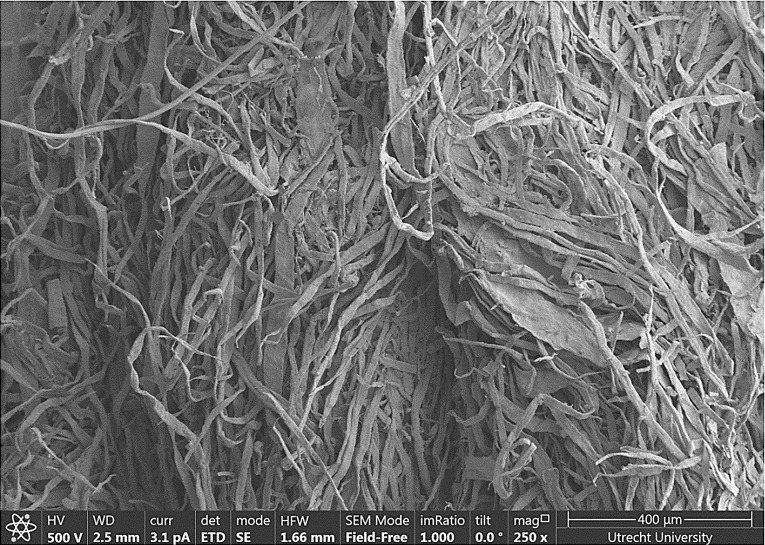
SEM image of paper sample’s surface

This study was performed on Ziegler Z-Plot 650, which is an uncoated filler-free fibrous layer. The sample had a thickness of $150\,\upmu \hbox {m}$ and surface roughness of about $35~\upmu \hbox {m}$. Samples were kept under room conditions ($21\,{^{\circ }}\hbox {C}$ and relative humidity of 88%) for 24 hours before performing an experiment. Physical properties of the paper are shown in Table 1. Cellulose fibers are the only component of the studied paper sample. The layer is made of fibers with various lengths and with a mean diameter of $20~\upmu \hbox {m}$. Fig. 1 shows a SEM image of surface of Ziegler paper.

### Imaging Methods

The pore space of the paper was imaged using a Zeiss Xradia Versa 520 micro-tomography scanner. The images had a voxel size (resolution) of $0.9~\upmu \hbox {m}$.

The water movement between the fibers of the paper was studied with a confocal laser scanning microscope (Nikon A1+ confocal microscopy, Tokyo, Japan). Water containing fluorescent salt (florescein sodium salt, Sigma Aldrich, Nederland) dissolved to a concentration of 1.5 gr/300 ml was used as the liquid. Water was provided by a syringe pump (11 Elite, Harvard Apparatus, UK) at a constant flow rate of 0.005 ml/min.

The fibers and ink were visualized using a combination of laser wave lengths of 405, 488, and 561nm. A 637-nm red diode laser was also used to detect fluorescent particle sin the ink. Optimization of imaging parameters yielded a clear distinguish between fibers and ink. Images of water flow in paper were captured using a 10$\times$ microscope objective, and the view domain was $1.8\times 1.8\hbox { mm}^{2}$.

**Fig. 2:**
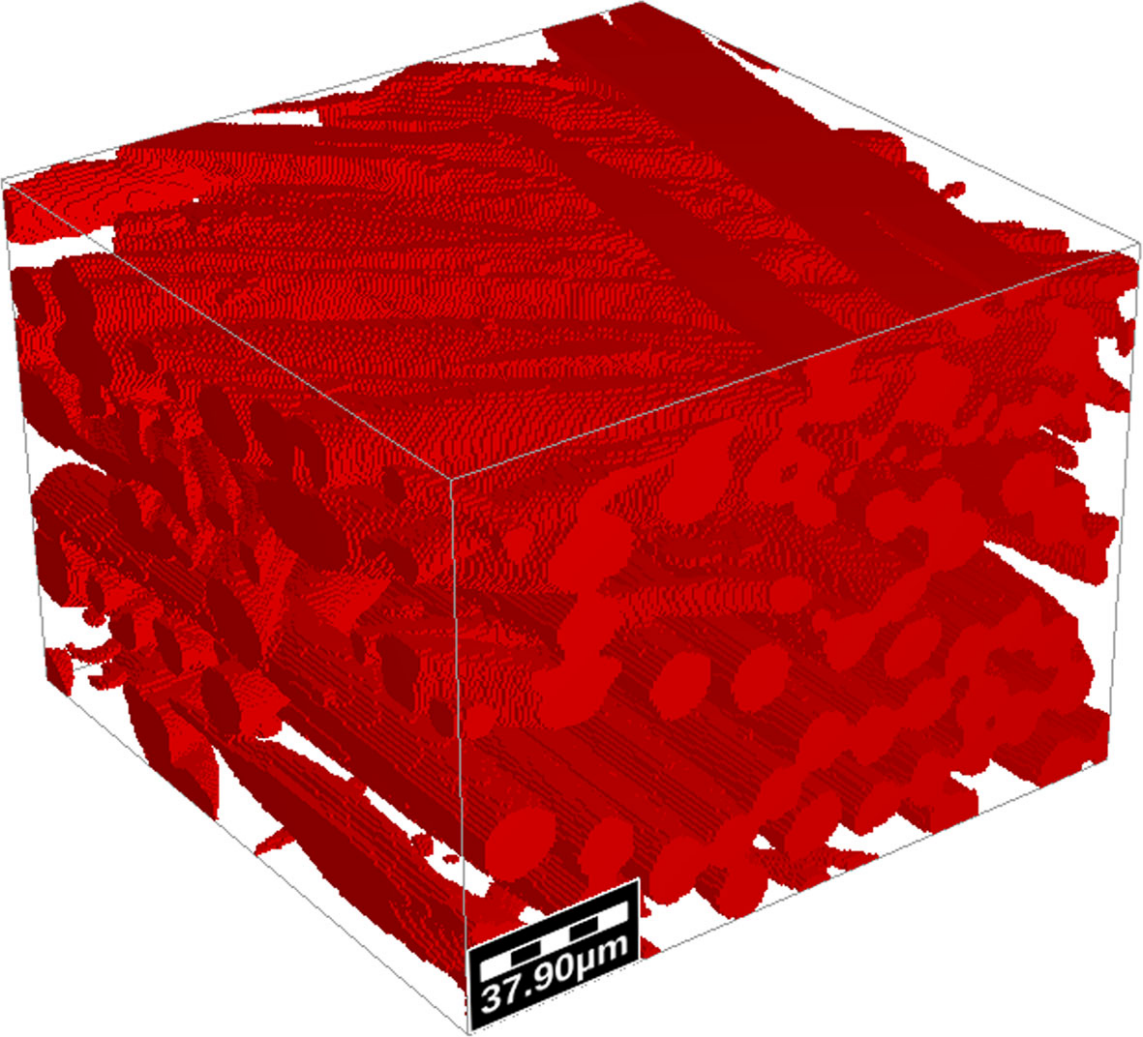
Binary three-dimensional domain of fibrous layer

### Image Analysis

$\upmu \hbox {CT}$ Images of the paper samples were analyzed to reconstruct 3D structure of the domain. All image analysis works were done using the software Avizo 9.0 Fire edition (FEI). A stack of two-dimensional high-resolution $(0.9~\upmu \hbox {m})$ images were first filtered using a Gaussian filter module to remove image noise. Then, using the interactive thresholding module (with intensity range of 32000–46500), the grayscale images were converted to binary images (Fig. 2).

In order to determine the volume of each pore, the separation and labeling modules were used. These modules separate the pores from each other and label them by a distance map-based method. Then, analysis of the labeled pores resulted in pore size distribution and porosity of the sample.

### Pore-Scale Simulation

Pore-scale flow properties were obtained using Pore Morphology method embedded in GeoDict software (Math2Market, Germany). Using this software, we could determine capillary pressure-saturation curves, permeability, and relative permeability. The calculation of saturation and capillary pressure was based on Young–Laplace equation. For an applied capillary pressure, which is the difference between wetting and non-wetting phase pressures, a pore radius is determined:

4 $$\begin{aligned} r=\frac{2\sigma }{P_\mathrm{c}}\cos \psi \end{aligned}$$

where $\sigma$ is surface tension, $\psi$ is the contact angle, $P_\mathrm{c}$ is the capillary pressure. The calculated pore radius determines the smallest pores (and throats) that will be filled with the wetting phase during imbibition, and the largest pores that will be filled with non-wetting phase during drainage (Hilpert and Miller 2001; Schulz et al. 2014; Aslannejad et al. 2017).

With the distribution of wetting and non-wetting phases inside the domain determined for a given capillary pressure, the saturation value could be calculated. Both primary imbibition and drainage curves were obtained.

To define, first Stokes equation was solved for steady-state flow of water within the pores at a certain applied pressure gradient. Then, the flow rate across the domain was calculated and Darcy’s law was used to obtain permeability (Cheng et al. 2013). The calculated permeability values in combination with porosity values were used in the determination of REV size. Relative permeability–saturation curve was obtained based on primary imbibition results as follows. As mentioned above, for any given imposed capillary pressure, the distribution of wetting and non-wetting phases within the pores was known. Then, a pressure gradient across the REV was imposed on the wetting phase and Stokes equation was solved to calculate flow rate. Therefore, for any imposed pressure, substitution of flow in Darcy’s law yielded effective permeability and consequently relative permeability values (Hilpert and Miller 2001; Cheng et al. 2013; Schulz et al. 2014).

### REV Size Determination

It is preferable to find the smallest REV size which provides representative average macroscale parameters of the domain. The REV size can be determined by calculation and comparison of values of a given hydraulic property for different REV sizes. It is the smallest domain size beyond which no difference in values of the property is found. For this study, ten different averaging domain sizes were selected. As the paper fibrous layer is anisotropic, liquid flow had to be considered in all three directions (*x*, *y*, and *z*). Due to the small number of pores in *z* (thickness) direction, all averaging domains were chosen to have the same thickness as the paper $(150\,\upmu \hbox {m})$ and their sizes were varied in lateral directions (*x*, *y*) from 50 to $1000~\upmu \hbox {m}$.

## Results and Discussion

### Pore Size Distribution

**Fig. 3:**
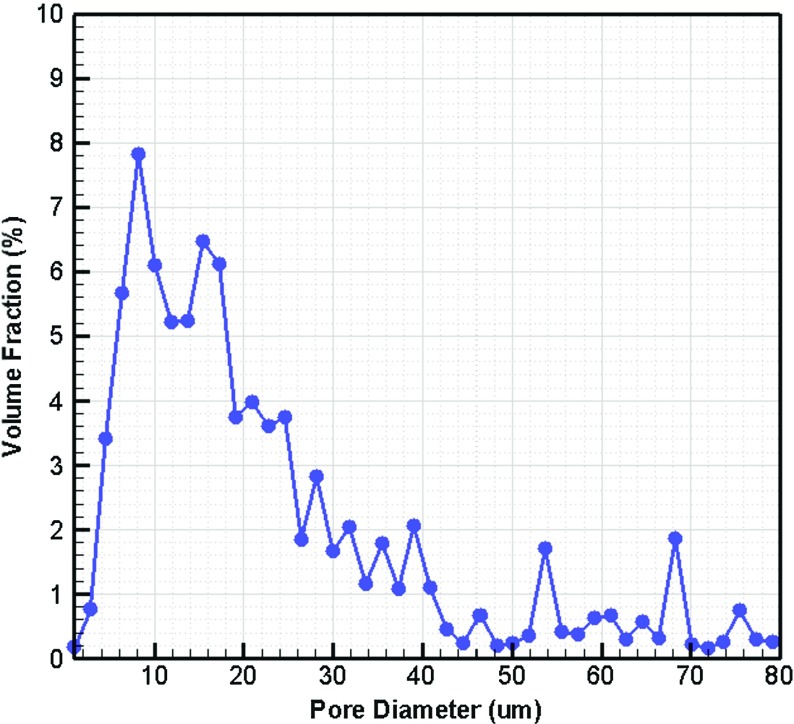
Pore size distribution of fibrous layer

The distance map values, obtained via image analysis, revealed pores and then pore size distribution of the layer (Fig. 3). The mean pore size was found to be $12~\upmu \hbox {m}$, which is in the same range as reported for similar materials by other researchers (Dodson and Sampson 1996).

### Determination of Hydraulic Properties

**Fig. 4:**
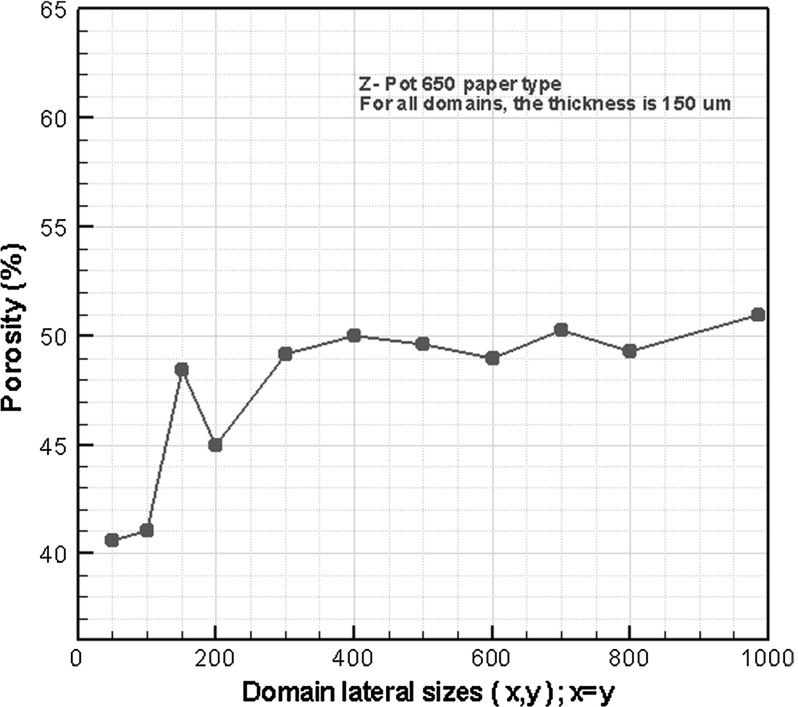
Porosity values for different domain sizes (thickness is constant and equal to 150 ${\upmu }$m)

The variation of porosity with the size of the averaging domain is shown in Fig. 4. Based on this figure, we concluded that for domain sizes of $400\times 400\times 150\,\upmu \hbox {m}^{3}$ and larger, the calculated porosity values are about 50% and do not vary significantly. For comparison, we recall that the REV size for the coating layer of paper was found to be $4\times 4\times 4\,\upmu \hbox {m}^{3}$ (Aslannejad et al. 2017).

**Fig. 5:**
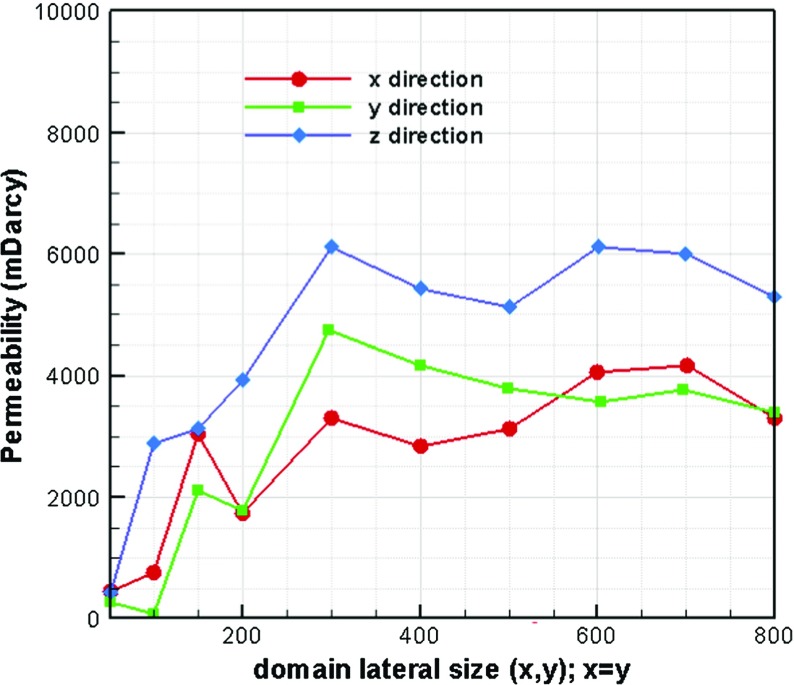
Permeability values for ten different domain sizes (*x*, *y*; $x=y$ and thickness is constant $150~\upmu \hbox {m}$ for all domains)

Increasing the domain size when calculating permeability resulted in higher permeability values. This is because larger domains yielded bigger mean pore sizes. We found that the correlation of the higher permeability value for the larger mean pore size just stands till reaching REV size. Permeability values for different domain sizes in three directions are plotted in Fig. 5. The variation of permeability value with changing domain size also gives an REV size of $400\,\upmu \hbox {m}$ $(400\times 400\times 150\,\upmu \hbox {m}^{3})$. In the case of a paper coating layer, the permeability value was found to be about 50 times smaller (Aslannejad et al. 2017).

**Fig. 6:**
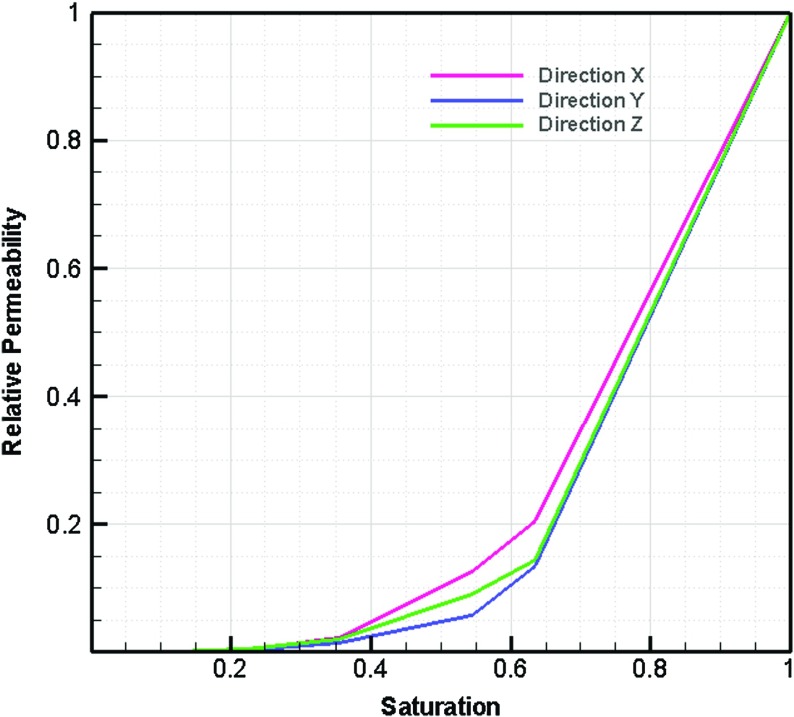
Relative permeability curve based on imbibition phase distribution

Permeability values in *z* direction (in thickness direction) were larger in comparison with lateral directions (see Fig. 5). This could be due to bigger pore sizes (and/ or) shorter pathways in connected pores in z direction. By fitting spheres in pores in three different directions, percolation paths were determined. The biggest particle size that could pass from one side to the opposite side in each direction was determined. Also, the path length divided by the length of a straight line in the selected direction was calculated. As shown in Table 2, larger particles could pass in z direction. This explains why we found a larger permeability value in z direction in comparison with the other two directions. Fig. 6 shows the relative permeability–saturation curve for the studied fibrous layer.

**Table 2:** Particle size and path length to determine percolation path

Direction	Particle diameter $(\upmu \hbox {m})$	Normalized path length$^\mathrm{a}$
*x*	13	1.31
*y*	13.2	1.16
*z*	21.3	1.7

$^\mathrm{a}\,$Path length divided by thickness of layer in specified direction

**Fig. 7:**
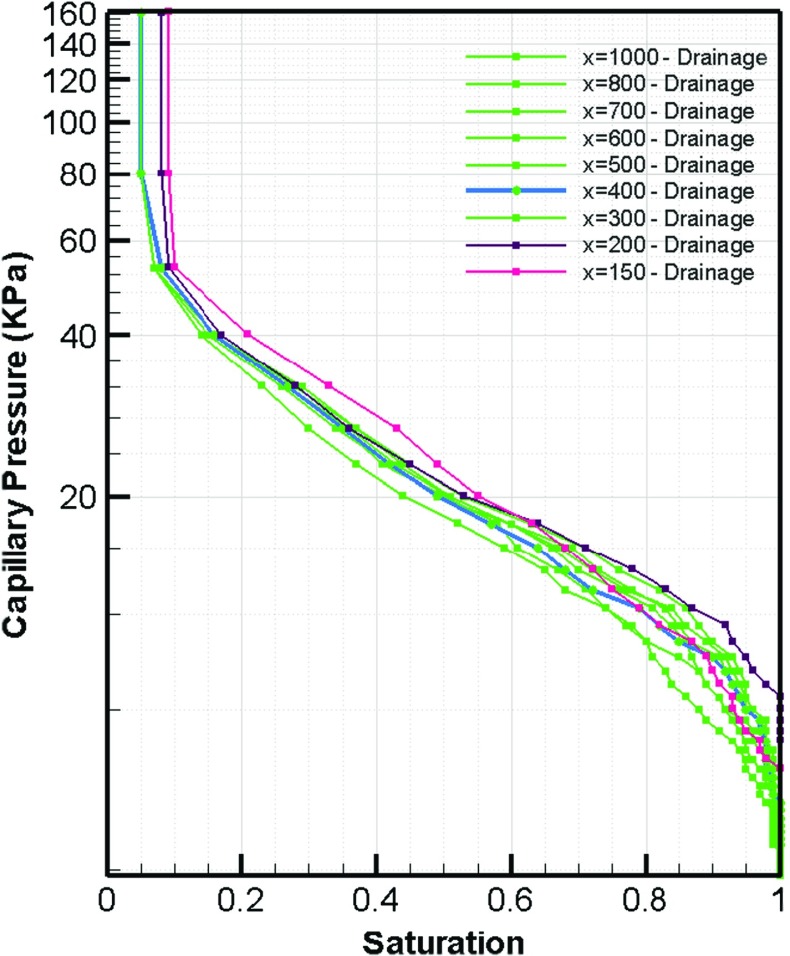
Drainage curves for selected domain sizes

**Fig. 8:**
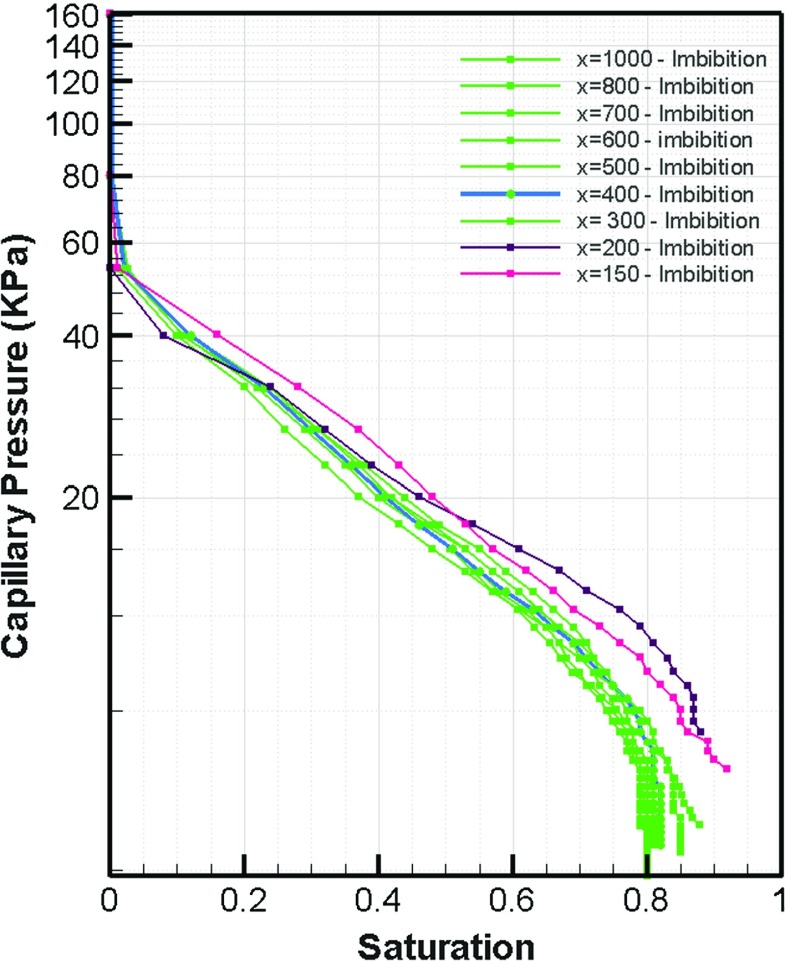
Imbibition curves for selected domain sizes

Plots of capillary pressure versus saturation  for different averaging domain sizes are shown in Figs. 7 and 8 for drainage and imbibition, respectively. As we can see, in each figure, the curves have a similar shape and range for domains bigger than $400~\upmu \hbox {m}$ in size. This is in good agreement with the REV size we obtained based on porosity and permeability results (Figs. 4 and 5).

**Fig. 9:**
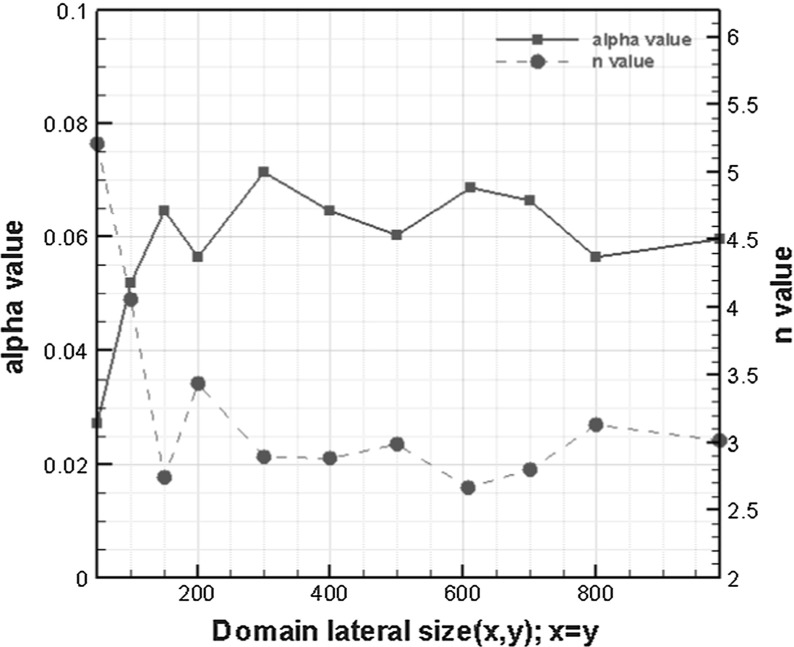
van Genuchten parameters ($\alpha$ and *n* values) for different domain sizes

We fitted van Genuchten (van Genuchten 1980) empirical equation to our simulated capillary pressure–saturation curves.

5 $$\begin{aligned}&\displaystyle S_e ({P_\mathrm{c}})=\left[ {1+\left( {\alpha P_\mathrm{c}} \right) ^{n}} \right] ^{-m} \end{aligned}$$

6 $$\begin{aligned}&\displaystyle S_e =\frac{S_w -S_\mathrm{ir} }{1-S_\mathrm{ir} -S_r } \end{aligned}$$

where $$S_e $$, $$S_r $$, $$S_\mathrm{ir} $$, and $$S_w $$ are effective saturation, air residual saturation, irreversible wetting phase saturation, and wetting phase saturation, respectively, $$P_{\mathrm{c}}$$ is the capillary pressure, and $$\alpha $$ and *n* are empirical parameters reflecting the average pore size and width of the pore size distribution, respectively. Commonly, we assume $$m=1{-}1/n$$. Fitting parameters based on primary imbibition diagrams for different domain sizes are shown in Fig. 9.

We can see that a minimum averaging domain size of $200\times 200\times 150\,\upmu \hbox {m}^{3}$ is sufficient for the capillary pressure–saturation curve. Thus, an REV size of $400\times 400\times 150\,\upmu \hbox {m}^{3}$ can be used for all properties. The parameter values for the selected REV size are given in Table 3.

### Directional Independence of Pc–S Curves

**Fig. 10:**
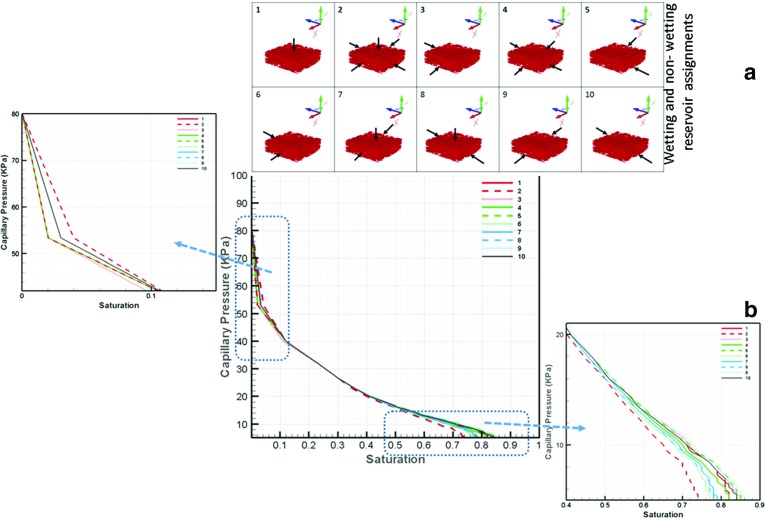
Wetting and non-wetting phase connection-side effect on capillary pressure–saturation curves; **a** Wetting phase connection-side configuration. **b**  *curves*

For the determination of capillary pressure–saturation curves, a non-wetting phase reservoir was assigned to one side of the domain and a wetting phase reservoir was fixed to another side. This meant that the invasion of the domain by a phase was forced to be in a given direction. The question was raised whether the selection of domain sides for wetting and non-wetting phase reservoirs had an effect on the resulting capillary pressure curve. Fig. 10a shows wetting and non-wetting phase reservoir connection sides, and Fig. 10b shows resulted  curves. It is evident that all curves coincide for the most part. There are some differences at high saturations. But, these are negligible for all practical cases. This result is commensurate with the fact that capillary pressure and saturation are scalar quantities and their relationship should be independent of direction.

**Table 3:** Values of the van Genuchten parameters of the Pc–S curve

	*n*	$\upalpha$ $(\hbox {kPa}^{-1})$	$S_{r}$	$S_{{ ir}}$
Primary imbibition	2.8	0.06	0.83	0.00

### Effect of Deformation of Layer on  Curves

**Fig. 11:**
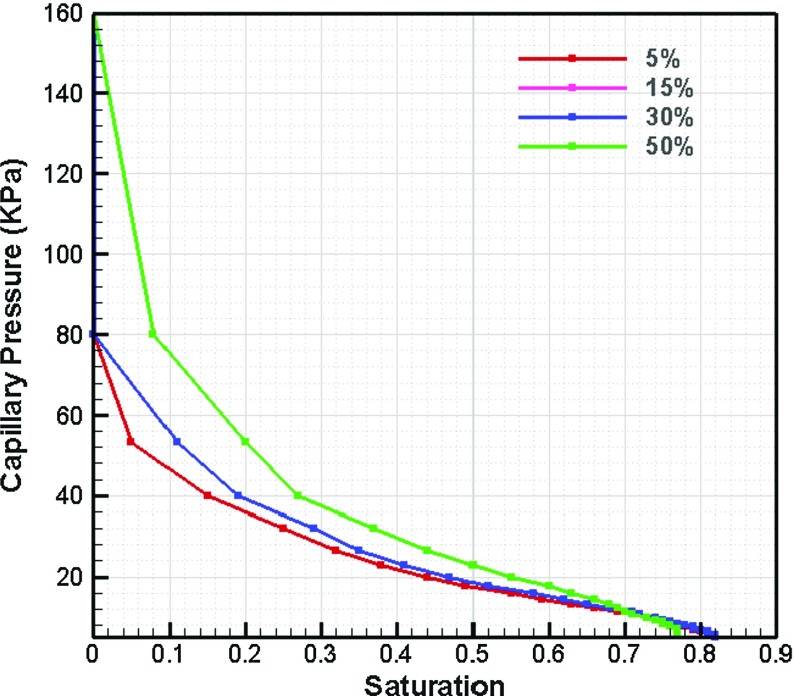
Effect of paper deformation on primary imbibition *curve*. Four different compression values are *plotted* with different *colors*

Deformation of paper is a relevant process in printing. Due to the compressibility of fibrous layer and possible pressing effect during printing, the pore space of paper may change in shape and size. To determine whether deformation may change macroscopic hydraulic properties of the layer, capillary pressure–saturation imbibition curves were obtained for different compression percentages (5, 15, 30, and 50%). Results are shown in Fig. 11. The effect of compression on pore space was simulated. A comparison of 50% reduces the layer thickness to half its original size. As compression decreases the pores sizes, it increases the entry capillary pressure value (as expected based on Eq. 1). Compression less than 30% had a negligible effect on capillary pressure–saturation curve.

### Visualization of Flow in Fibrous Layer

**Fig. 12:**
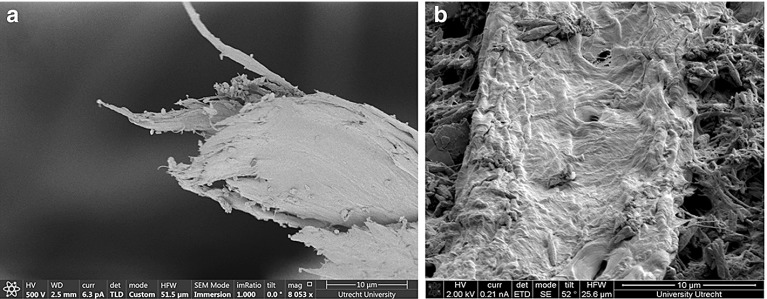
Visualization of individual fibers using electron microscopy: **a** bundle of fibrils, **b** surface of individual fiber

Water penetration into the paper fibrous layer is different from water movement into a granular porous material. In case of granular materials, water moves into the pores and wets the surface of pores simultaneously. But in a fibrous layer made of cellulose fibers, in addition to water movement inside pores which are made between fibers, individual fibers also have a rule in movement of water. The fibers are known to be made of bundle of fibrils. A bundle of fibrils is made of individual fibrils with a dimeter of about 2 nm. Fig. 12a shows a SEM image of one single fiber, in which few fibril bundles are visible. The cellulose material of the fibril is known to be strongly hydrophilic (see e.g. Hirn and Schennach 2015). So, the empty spaces between fibrils inside a bundle (about a few Nano meters) are expected to imbibe water strongly. In addition, SEM images show that the fiber surface has submicron roughness (Fig. 12b), forming micro-channels that can have a high capillary action.

**Fig. 13:**
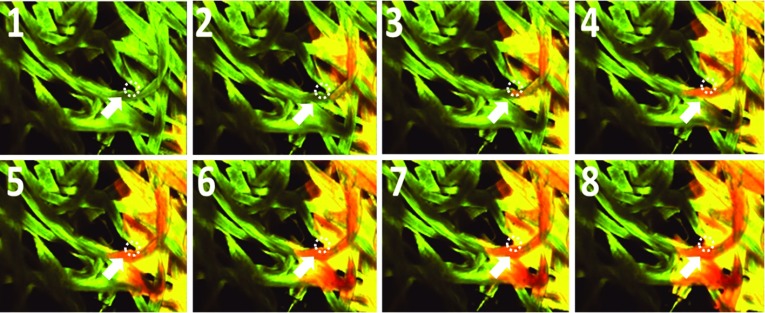
Water movement in fibrous paper layer

Indeed, confocal laser scanning microscopy of water movement in the fibrous layer provides a sequence of images, during rime (interval 3s), showing how water moves inside the layer. In Fig. 13, fibers are imaged in green color and water (containing fluorescent particles) is shown in red. The right-hand side of the fibrous layer is connected to water reservoir. Based on the results, water first wets the surface of fibers and perhaps fills its internal pores before filling the pores between fibers. We see in Fig. 13 that two wetting fronts develop: a very irregular zigzag front where water invades individual fibers and a relatively regular front moving behind and filling the pores.

Comparing liquid flow in granular material and fibrous layer, in first case, liquid fills pores and follows their connectivity. In addition, wetting the pore surface and imbibition of the pore occurs at the same time. Nevertheless, in case of fibrous layer, liquid wets surface of fibers and moves on surface of fibers, fingering-like front. A liquid front behind the fingering front moves and fills the pores between fibers.

**Fig. 14:**
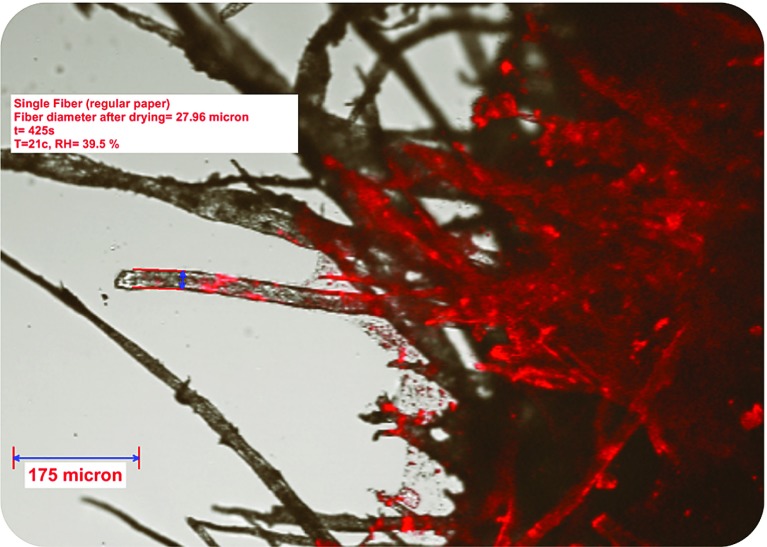
Swollen of a single cellulose fiber

Cellulose fibers absorb water and swell. Consequently, the diameter of fibers increases. In turn, this causes deformation of the layer. In Fig. 14, a swollen single fiber is shown. The fiber diameter before wetting was $26.74~\upmu \hbox {m}$. When water arrived to the selected area of fiber, it caused swelling and fiber diameter reached $32.87~\upmu \hbox {m}$ in 50 s. Then, after drying for 280 s, the diameter decreased to a value of $27.96\,\upmu \hbox {m}$. This shows that the change in fiber diameter is not completely reversible.

## Conclusion

In this work, pore-scale imaging and modeling of fluid flow in a fibrous layer were performed to determine hydraulic parameters of the layer. Imaging of the layer was done by $\upmu \hbox {CT}$ and then, by means of image analysis methods, 3D reconstruction of the layer was extracted. By comparison of porosity and permeability values as well as  curves for various domain sizes, the REV size of the layer was found to be $400\times 400\times 150\,(\upmu \hbox {m}^{3})$, with the layer thickness being $150~\upmu \hbox {m}$. The porosity and mean pore size values for the REV were found to be 50% and $12~\upmu \hbox {m}$, respectively. Checking the percolation path for the layer in three different directions, we found that bigger pores connection exists in z direction (the thickness direction), but it has shorter normalized path length in y direction. No directional dependence for $P_{\mathrm{c}}$-s curves was found.

In order to check the impact of layer deformation (compression during printing) on  curve, four different compression values were chosen and checked. That was concluded that compression less than 30% does not has any significant effect on  curves.

Finally, dynamic visualization of water flow in fibrous layer showed that water moves along and into the fiber first and then fills the pore between them. Swelling of individual fibers was measured and was found to be partially irreversible.
